# Multi technique amalgamation for enhanced information identification with content based image data

**DOI:** 10.1186/s40064-015-1515-4

**Published:** 2015-12-01

**Authors:** Rik Das, Sudeep Thepade, Saurav Ghosh

**Affiliations:** Department of Information Technology, Xavier Institute of Social Service, Dr. Camil Bulcke Path (Purulia Road), P.O. Box 7, Ranchi, 834001 Jharkhand India; Pimpri Chinchwad College of Engineering, Akrudi, Sec-26,Pradhikaran, Nigdi, Pune, 411033 Maharashtra India; A.K. Choudhury School of Information Technology, University of Calcutta, 92, APC Road, Kolkata, 700009 West Bengal India

**Keywords:** Image classification, Image retrieval, Otsu’s threshold, Slant transform, Morphological operator, Fusion, *t* test

## Abstract

Image data has emerged as a resourceful foundation for information with proliferation of image capturing devices and social media. Diverse applications of images in areas including biomedicine, military, commerce, education have resulted in huge image repositories. Semantically analogous images can be fruitfully recognized by means of content based image identification. However, the success of the technique has been largely dependent on extraction of robust feature vectors from the image content. The paper has introduced three different techniques of content based feature extraction based on image binarization, image transform and morphological operator respectively. The techniques were tested with four public datasets namely, Wang Dataset, Oliva Torralba (OT Scene) Dataset, Corel Dataset and Caltech Dataset. The multi technique feature extraction process was further integrated for decision fusion of image identification to boost up the recognition rate. Classification result with the proposed technique has shown an average increase of 14.5 % in Precision compared to the existing techniques and the retrieval result with the introduced technique has shown an average increase of 6.54 % in Precision over state-of-the art techniques.

## Background

Recent years have witnessed the digital photo-capture devices as a ubiquity for the common mass (Raventós et al. 2015). The low cost storage, increasing computer power and ever accessible internet have kindled the popularity of digital image acquisition. Efficient indexing and identification of image data from these huge image repositories has nurtured new research challenges in computer vision and machine learning (Madireddy et al. 2014). Automatic derivation of sematically-meaningful information from image content has become imperative as the traditional text based annotation technique has revealed severe limitations to fetch information from the gigantic image datasets (Walia et al. 2014). Conventional techniques of image recognition were based on text or keywords based mapping of images which had limited image information. It was dependent on the perception and vocabulary of the person performing the annotation. The manual process was highly time consuming and slow in nature. The aforesaid limitations have been effectively handled with content based image identification which has been exercised as an effective alternative to the customary text based process (Wang et al. 2013). The competence of the content based image identification technique has been dependent on the extraction of robust feature vectors. Diverse low level features namely, color, shape, texture etc. have constituted the process of feature extraction. However, an image comprises of number of features which can hardly be defined by a single feature extraction technique (Walia et al. 2014). Therefore, three different techniques of feature extraction namely, feature extraction with image transform, feature extraction with image morphology and feature extraction with image binarization have been proposed in this paper to leverage fusion of multi-technique feature extraction. The recognition decision of three different techniques was further integrated by means of *Z* score normalization to create hybrid architecture for content based image identification. The main contribution of the paper has been to propose fusion architecture for content based image recognition with novel techniques of feature extraction for enhanced recognition rate.

The research objectives have been enlisted as follows:

- Reducing the dimension of feature vectors.
- Successfully implementing fusion based method of content based image identification.
- Statistical validation of research results.
- Comparison of research results with state-of-the art techniques.

Three different techniques of feature extraction using image binarization, image transforms and morphological operators have been combined to develop fusion based architecture for content based image classification and retrieval. Hence, it is in correlation with research on binarization based feature extraction, transform based feature extraction and morphology based feature extraction from images. It is also in connection with research on multi technique fusion for content based image identification. Therefore, the following four subsections have reviewed some contemporary and earlier works on these four topics.

### Feature extraction using image transform

Change of domain of the image elements has been carried out by using image transformation to represent the image by a set of energy spectrum. An image can be represented as series of basis images which can be formed by extrapolating the image into a series of basis functions (Annadurai and Shanmugalakshmi 2011). The basis images have been populated by using orthogonal unitary matrices as image transformation operator. This image transformation from one representation to another has advantages in two aspects. An image can be expanded in the form of a series of waveforms with the use of image transforms. The transformation process has been helpful to differentiate the critical components of image patterns and in making them directly accessible for analysis. Moreover, the transformed image data has a compact structure useful for efficient storage and transmission. The aforesaid properties of image transforms facilitate radical reduction of feature vector dimension to be extracted from the images. Diverse techniques of feature extraction has been proposed by exploiting the properties of image transforms to extract features from images using fractional energy coefficient (Kekre and Thepade 2009; Kekre et al. 2010). The techniques have considered seven image transforms and fifteen fractional coefficients sets for efficient feature extraction. Original images were divided into subbands by using multiple scales Biorthogonal wavelet transform and the subband coefficients were used as features for image classification (Prakash et al. 2013). The feature spaces were reduced by applying Isomap-Hysime random anisotropic transform for classification of high dimensional data (Luo et al. 2013).

### Image binarization techniques for feature extraction

Feature extraction from images has been largely carried out by means of image binarization. Appropriate threshold selection has been imperative for execution of efficient image binarization. Nevertheless, various factors including uneven illumination, inadequate contrast etc. can have adverse effect on threshold computation (Valizadeh et al. 2009). Contemporary literatures on image binarization techniques have categorized three different techniques for threshold selection namely, mean threshold selection, local threshold selection and global threshold selection to deal with the unfavourable influences on threshold selection. Enhanced classification results have been comprehended by feature extraction from mean threshold and multilevel mean threshold based binarized images (Kekre et al. 2013; Thepade et al. 2013a, b). Eventually, it has been identified that selection of mean threshold has not dealt with the standard deviation of the gray values and has concentrated only on the average which has prevented the feature extraction techniques to take advantage of the spread of data to distinguish distinct features. Therefore, image signature extraction was carried out with local threshold selection and global threshold selection for binarization, as the techniques were based on calculation of both mean and standard deviation of the gray values (Liu 2013; Yanli and Zhenxing 2012; Ramírez-Ortegón and Rojas 2010; Otsu 1979; Shaikh et al. 2013; Thepade et al. 2014a).

### Use of morphological operators for feature extraction

Commercial viability of shape feature extraction has been well highlighted by systems like Image Content (Flickner et al. 1995), PicToSeek (Gevers and Smeulders 2000). Two different categorization of shape descriptors namely, contour-based and region-based descriptors have been elaborated in the existing literatures (Mehtre et al. 1997; Zhang and Lu 2004). Emphasize of the contour based descriptors has been on boundary lines. Popular contour-based descriptors have embraced Fourier descriptor (Zhang and Lu 2003), curvature scale space (Mokhtarian and Mackworth 1992), and chain codes (Dubois and Glanz 1986). Feature extraction from complex shapes has been well carried out by means of region-based descriptors, since the feature extraction has been performed from whole area of object (Kim and Kim 2000).

### Fusion methodologies and multi technique feature extraction

Information recognition with image data has utilized the features extracted by means of diverse extraction techniques to harmonize each other for enhanced identification rate. Recent studies in information fusion have categorized the methodologies typically into four classes, namely, early fusion, late fusion, hybrid fusion and intermediate fusion. Early fusion combines the features of different techniques and produces it as a single input to the learner. The process inherently increases the size of feature vector as the concentrated features easily correspond to higher dimensions. Late fusion applies separate learner to each feature extraction technique and fuses the decision with a combiner. Although it offers scalability in comparison to early fusion, still, it cannot explore the feature level correlations, since it has to make local decisions primarily. Hybrid fusion makes a mix of the two above mentioned techniques. Intermediate fusion integrates multiple features by considering a joint model for decision to yield superior prediction accuracy (Zhu and Shyu 2015). Color and texture features were extracted by means of 3 D color histogram and Gabor filters for fusion based image identification. The space complexity of the feature was further reduced by using genetic algorithm which has also obtained the optimum boundaries of numerical intervals. The process has enhanced semantic retrieval by introducing feature selection technique to reduce memory consumption and to decrease retrieval process complexity (ElAlami 2011). Local descriptors based on color and texture was calculated from Color moments and moments on Gabor filter responses. Gradient vector flow fields were calculated to capture shape information in terms of edge images. The shape features were finally depicted by invariant moments. The retrieval decisions with the features were fused for enhanced retrieval performance (Hiremath and Pujari 2007). Feature vectors comprising of color histogram and texture features based on a co-occurrence matrix were extracted from HSV color space to facilitate image retrieval (Yue et al. 2011). Visually significant point features chosen from images by means of fuzzy set theoretic approach. Computation of some invariant color features from these points was performed to gauge the similarity between images (Banerjee et al. 2009). Recognition process was boosted up by combining color layout descriptor and Gabor texture descriptor as image signatures (Jalab 2011). Multi view features comprising of color, texture and spatial structure descriptors have contributed for increased retrieval rate (Shen and Wu 2013). Wavelet packets and Eigen values of Gabor filters were extracted as feature vectors by the authors in (Irtaza et al. 2013) for neural network architecture of image identification. The back propagation neural network was trained on sub repository of images generated from the main image repository and utilizes the right neighbourhood of the query image. This kind of training was aimed to insure correct semantic retrieval in response to query images. Higher retrieval results have been apprehended with intra-class and inter-class feature extraction from images (Rahimi and Moghaddam 2013). In (ElAlami 2014), extraction of color and texture features through color co-occurrence matrix (CCM) and difference between pixels of scan pattern (DBPSP) has been demonstrated and an artificial neural network (ANN) based classifier was designed. In (Subrahmanyam et al. 2013), content-based image retrieval was carried out by integrating the modified color motif co-occurrence matrix (MCMCM) and difference between the pixels of a scan pattern (DBPSP) features with equal weights. Fusion of semantic retrieval results obtained by capturing colour, shape and texture with the color moment (CMs), angular radial transform descriptor and edge histogram descriptor (EHD) features respectively had outclassed the Precision values of individual techniques (Walia et al. 2014). Six semantics of local edge bins for EHD were considered which included the vertical and the horizontal edge (0,0), 45^°^ edge and 135^°^ edge of sub-image (0,0), non directional edge of sub-image (0,0) and vertical edge of sub-image at (0,1). Color histogram and spatial orientation tree has been used for unique feature extraction from images for retrieval purpose (Subrahmanyam et al. 2012).

## Methods

Three different techniques of feature extraction have been introduced in this work namely, feature extraction with image binarization, feature extraction with image transform and feature extraction with morphological operator. However, there are popular feature extraction techniques like GIST descriptor which has much greater feature dimension compared to the proposed techniques in the work. GIST creates 32 feature maps of same size by convolving the image with 32 Gabor filters at 4 scales, 8 orientations (Douze et al. 2009). It averages the feature values of each region by dividing each feature map into 16 regions. Finally, it concatenates the 16 average value of all 32 feature maps resulting in 16 × 32 = 512 GIST descriptor. On the other hand, our approach has generated a feature dimension of 6 from each of the binarization and morphological technique. Feature extraction by applying image transform has yielded a feature size of 36. On the whole, the feature size for the fusion based classifier was (6 + 36 + 6 = 48) which is far less than GIST and has much lesser computational overhead. Furthermore, fusion based architecture for classification and retrieval have been proposed for enhanced identification rate of image data. Each of the techniques of feature extraction as well as the methods for fusion based architecture of classification and retrieval has been discussed in the following four subsections and the description of datasets has been given in the fifth subsection.

### Feature extraction with image binarization

**Fig. 1:**
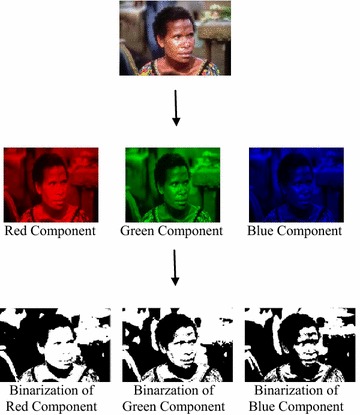
Binarization using Otsu’s Threshold selection

Initially, the three color components namely, Red (R), Green (G) and Blue (B) were separated in each of the test images. A popular global threshold selection method named Otsu’s method has been applied separately on each of the color components for binarization as in Fig. 1. The above mentioned thresholding method has been largely used for document image binarzation. Otsu’s technique has been operated directly on the gray level histogram which has made it fast executable. It has been efficient to remove redundant details from the image to bring out the necessary image information. The method has been considered as a non-parametric method which has considered two classes of pixels, namely, the foreground pixels and the background pixels. It has calculated the optimal threshold by using the within-class variance and between-class variance. The separation was carried out in such a way so that their combined intra-class variance is minimal (Otsu 1979; Shaikh et al. 2013). Comprehensive investigation has been carried out for the threshold that minimizes the intra-class variance represented by the weighted sum of variances of the two classes of pixels for each of the three color components.

The weighted within-class variance has been given in Eq. 1.

1 $$\sigma_{w}^{2} (t) = q_{1} (t)\sigma_{1}^{2} (t) + q_{2} (t)\sigma_{2}^{2} (t)$$

*q*_1_(*t*) = ∑ _*i*=0_^*t*^*P*(*i*) where the class probabilities of different gray level pixels were estimated as shown in Eqs. 2 and 3:

2 $$q_{1} (t) = \sum\limits_{i = 0}^{t} {p(i)}$$

3 $$q_{2} (t) = \sum\limits_{i = t + 1}^{255} {P(i)}$$

The class means were given as in Eqs. 4 and 5:

4 $$\mu_{1} (t) = \sum\limits_{i = 0}^{t} {\frac{i*P(i)}{{q_{1} (t)}}}$$

5 $$\mu_{2} (t) = \sum\limits_{i = t + 1}^{255} {\frac{i*P(i)}{{q_{2} (t)}}}$$

Total variance (*σ*^2^) = Within-class variance (*σ*_*w*_^2^(*t*)) + Between-class Variance(*σ*_*b*_^2^(*t*)).

Since the total variance was constant and independent of *t*, the effect of changing the threshold was purely to shift the contributions of the two terms back and forth. Between-class variance has been given in Eq. 6

6 $$\sigma_{b}^{2} (t) = q_{1} (t)[1 - q_{1} (t)][\mu_{1} (t) - \mu_{2} (t)]^{2}$$

Thus, minimizing the within-class variance was the same as maximizing the between-class variance.

Binarization of the test images was carried out using the Otsu’s local threshold selection method. The process has been repeated for all the three color components to generate bag of words model (BoW) of features. Conventional BoW model has been based on SIFT algorithm which has a descriptor dimension of 128 (Zhao et al. 2015). Therefore, for three color components the dimension of the descriptor would have been 128 × 3 = 384. The size for SIFT descriptor has been huge and it has predestined problem for information losses and omissions as it has been found suitable only for the stability of image feature point extraction and description. Furthermore, the generated SIFT descriptors has to be clustered by *k* means clustering which has been based on allocation of cluster members by means of comparing squared Euclidian distance. The clustering process has been helpful to generate codewords for codebook generation which has been the final step of BoW. Process of *k* means clustering has huge computational overhead for calculating the squared *Euclidian* distance which eventually slows down the BoW generation. Hence, in our approach, the grey values higher than the threshold was clustered in higher intensity group and the grey values lower than the cluster was clustered in the lower intensity group. The mean of the two groups were calculated to formulate the codewords of higher intensity feature vectors and the lower intensity feature vectors respectively. Thus, each color component of a test image has been mapped to two codewords of higher intensity and lower intensity respectively. This has generated of codebook of size (3 × 2 = 6) for each image.

The algorithm for feature extraction has been stated in Algorithm 1 as follows:

**Algorithm 1**

### Feature extraction using image transform

**Fig. 2:**
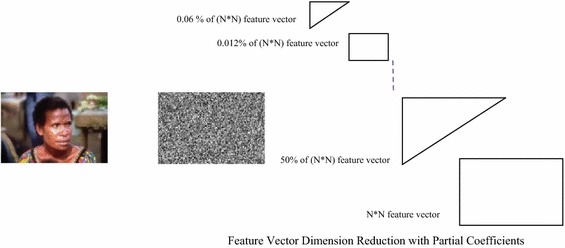
Feature extraction by applying image transform

Transforms convert spatial information to frequency domain information, where certain operations are easier to perform. Energy compaction property of transforms has the capacity to pack large fraction of the average energy into a few components. This has led to faster execution and efficient algorithm design. Image transforms has the property to convert the spatial domain information of an image to frequency domain information, where certain operations are easier to perform. For example, convolution operation can be reduced to matrix multiplication in frequency domain. It has the characteristic of energy compaction which ensures that a large fraction of the average energy of the image remains packed into a few components. This property has led to faster execution and efficient algorithm design by drastic reduction of feature vector size which is achieved by means of discarding insignificant transform coefficients as in Fig. 2. The approach has been implemented by applying slant transform on each of the Red (R), Green (G) and Blue (B) color component of the image for extraction of feature vectors with smaller dimension. Slant transform has reduced the average coding of a monochrome image from 8 bits/pixel to 1 bit/pixel without seriously degrading the image quality. It is an orthogonal transform which has also reduced the coding of color images from 24–2 bits/pixel (Pratt et al. 1974). Slant transform matrices are orthogonal and it holds all real components. Hence, it has much less computational overhead compared to discrete Fourier transform. Slant transform is an unitary transform and follows energy conservation. It tends to pack a large fraction of signal energy into a few transform coefficients which has a significant role in reducing the feature vector for the image. Let [*F*] be an *N × N* matrix of pixel values of an image and let [*f*_*i*_] be an *N* *×* *1* vector representing the *i*th. column of [*F*]. One dimensional transform of the *i*th. image line can be given by

$$\left[ {f_{i} } \right] = \left[ S \right]\left[ {f_{i} } \right]$$

[*S*] = *N × N* unitary slant matrix.

A two dimensional slant transform can be performed by sequential transformations of row and column of [*F*] and the forward and inverse transform can be expressed as in Eqs. 7 and 8.

7 $$\left[ \Im \right] = \left| S \right|\left[ F \right]\left[ S \right]^{T}$$

8 $$\left[ F \right] = \left[ S \right]^{T} \left[ \Im \right]\left[ S \right]$$

A transform operation can be conveniently represented in a series. The two dimensional forward and inverse transform in series form can be represented as in Eqs. 9 and 10

9 $$\Im ( {u, v}) = \sum_{j= {{1}}}^{N} \sum _{k = {{1}}}^{N} F ( {j, k} )S ( {u, j} )S ( {k, v})$$

10 $$F\left( {j, k} \right) = \sum _{u={{1}}}^{N} \sum _{v={ {1}}}^{N} \Im \left( {u, v} \right)S\left( {j, u} \right)S\left( {v, k} \right)$$

The algorithm for feature extraction using slant transform has been given in Algorithm 2.

**Algorithm 2**

Here the features were extracted in the form of visual words. Visual words have been defined as a small patch of image which can carry significant image information. The energy compaction property of Slant transform has condensed noteworthy image information in a block of 12 elements for an image of dimension (256 × 256). Thus, the feature vector extracted with slant transform was of size 12 for each color component which has given the dimension of feature vector as 36 (12 × 3 = 36) for three color components in each test image.

### Feature extraction with morphological operator

Human perception has largely been governed by shape context. It has been helpful to recover the point correspondences from an image which has considerable contribution in feature vector formation. A variant of gray scale opening and closing operations has been termed as the top-hat transformation that has been instrumental in producing only the bright peaks of an image (Sridhar 2011). It has been termed as the *peak detector* and its working process has been given as follows:

1. Apply the gray scale opening operation to an image.
2. Peak = original image—opened image.
3. Display the peak.
4. Exit.

**Fig. 3:**
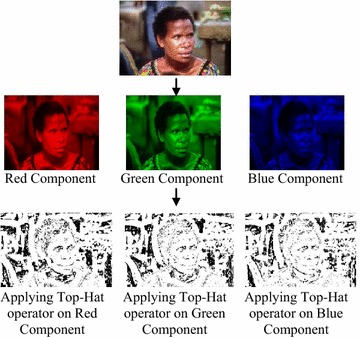
Effect of applying morphological operator

The top-hat transform technique was applied on each color component Red (R), Green (G) and Blue (B) of the test images for feature extraction using morphological operator as in Fig. 3. After applying the tophat operator, the pixels designated as the foreground pixels were grouped in one cluster and were calculated with mean and standard deviation to formulate the higher intensity feature vector. Similar process was followed with the pixels designated as the background pixels to calculate the lower intensity feature vector. The feature vector extraction process has followed the bag of words (BoW) methodology which has generated codewords from the cluster of foreground and background pixels by calculating the mean and the standard deviation of both the clusters and adding the two. Hence, codebook size for each color component was two which have yielded a dimension of 6 (3 × 2 = 6) on the whole for the codebook generated for three color components for each test image.

The algorithm for feature extraction using morphological operator has been given in Algorithm 3.

**Algorithm 3**

### Similarity measures

Determination of image similarity measures was performed by evaluating distance between set of image features. Higher similarity has been characterized by shorter distance (Dunham 2009). A fusion based classifier, an artificial neural network (ANN) classifier and a support vector machine (SVM) classifier was used for the purpose. Each of the classifier types has been discussed in the following sections:

### Fusion based classifier

Three different distance measures, namely, city block distance, Euclidian distance and mean squared error (MSE) distance metric was considered to compute the distance between query image *Q* and database image *T* as in Eqs. 11, 12 and 13

11 $$D_{cityblock} = \sum _{i - 1}^{n} \left| {Q_{i} - D_{i} } \right|$$

12 $$D_{euclidian} = \sqrt {\sum\limits_{i = 1}^{n} {(Q_{i} - D_{i} )^{2} } }$$

13 $$D_{MSE} = \frac{1}{n}\sum\limits_{i = 1}^{n} {\left( {Q_{i} - D_{i} } \right)^{2} }$$

where, *Q*_*i*_ is the query image and *D*_*i*_ is the database image.

Data standardization technique was followed to standardize the calculated distances for the individual techniques with *Z* score normalization which was based on mean and standard deviation of the computed values as in Eq. 14. The normalization process has been implemented to avoid dependence of the classification decision on a feature vector with higher values of attributes which have the possibilities to have greater effect or “weight.” The process has normalized the data within a common range such as [−1, 1] or [0.0, 1.0].

14 $$dist_{n} = \frac{{dist_{i} - \mu }}{\sigma }$$

where, *µ* is the mean and *σ* is the standard deviation.

Further, the final distance was calculated by adding the weighted sum of individual distances. The weights were calculated from the precision values of corresponding techniques. Finally, the image was classified based on the class majority of k nearest neighbors [Sridhar 2011] where value of k was

$$k \le \sqrt {number..of..training..ins\tan ces}.$$

The classified image was forwarded for retrieval purpose. The image was a classified query and has searched for similar images only within the class of interest. Ranking of the images was done with Canberra Distance measure as in Eq. 15 and top 20 images were retrieved.

15 $$D_{canberra} = \sum\limits_{i = 1}^{n} {\frac{{\left| {Q_{i} - D_{i} } \right|}}{{\left| {Q_{i} } \right| + \left| {D_{i} } \right|}}}$$

where, *Q*_*i*_ is the query image and *D*_*i*_ is the database image.

**Fig. 4:**
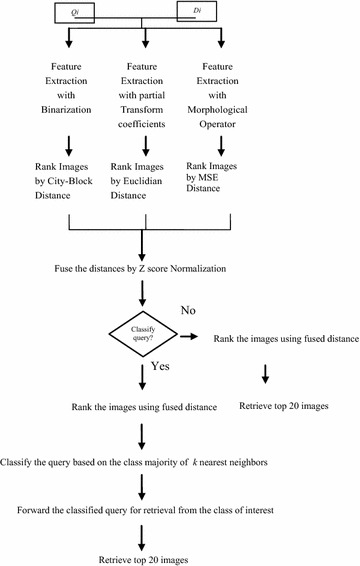
Fusion technique for image identification

The process of fusion based classification and then retrieval with classified query has been illustrated in Fig. 4.

### Artificial neural network (ANN) classifier

**Fig. 5:**
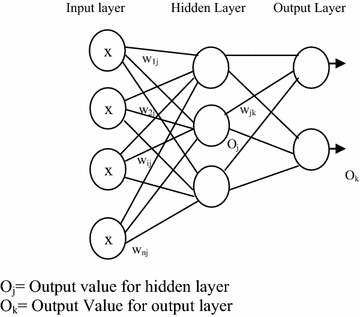
Multilayer perceptron (MLP)

The set of input features from images were mapped to an appropriate output by a feed forward Neural Network Classifier known as Multilayer Perceptron (MLP) as shown in Fig. 5 (Alsmadi et al. 2009).

The back propagation technique of multi layer perceptron has a significant role in supervised learning procedure. The network has been trained for optimization of classification performance by using the procedure of back propagation. For each training tuple, the weights were modified so as to minimize the mean squared error between the network prediction and the target value. These modifications have been made in the backward direction through each hidden layer down to the first hidden layer. The input feature vectors have been fed to the input units which comprised the input layer. The number of input units has been dependent on the summation of the number of attributes in the feature vector dataset and the bias node. The subsequent layer has been the hidden layer whose number of nodes has to be determined by considering the half of the summation of the number of classes and the number of attributes per class. The inputs that have passed the input layer have to be weighted and fed simultaneously to the hidden layer for further processing. Weighted output of the hidden layer was used as input to the final layer which has been named as the output layer. The number of units in the output layer has been denoted by the number of class labels. The feed forward property of this architecture does not allow the weights to cycle back to the input units.

### Support vector machine (SVM) classifier

**Fig. 6:**
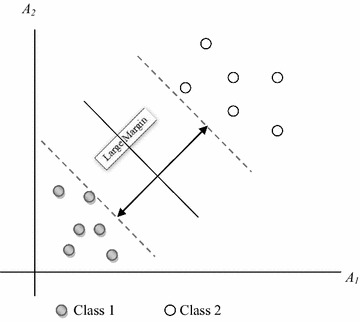
Structure of hyperplane in SVM

SVM transforms original training data to higher dimension by using nonlinear mapping. Optimal separating hyperplane has to be searched by the algorithm within this new dimension. Data from two different classes can readily be separated by a hyperplane by means of an appropriate nonlinear mapping to a sufficiently high dimension as in Fig. 6.

SVM has searched for the maximum separating hyperplane as shown in Fig. 6. The support vectors have been shown with thicker borders.

The algorithm was implemented using sequential minimal optimization (SMO) (Keerthi et al. 2001). The operating principle of SMO has been to select two Lagrange multipliers as the multipliers must obey a linear equality constraint. The two selected Lagrange multipliers jointly optimize to find the optimal value for these multipliers and updates the SVM to reflect the new optimal values.

### Datasets used

Four different datasets namely Wang dataset, Oliva and Torralba (OT-Scene) dataset, Corel dataset and Caltech Dataset was used for the content based image recognition purpose. Each of the datasets has been described in the following subsections.

### Wang’s dataset

**Fig. 7:**
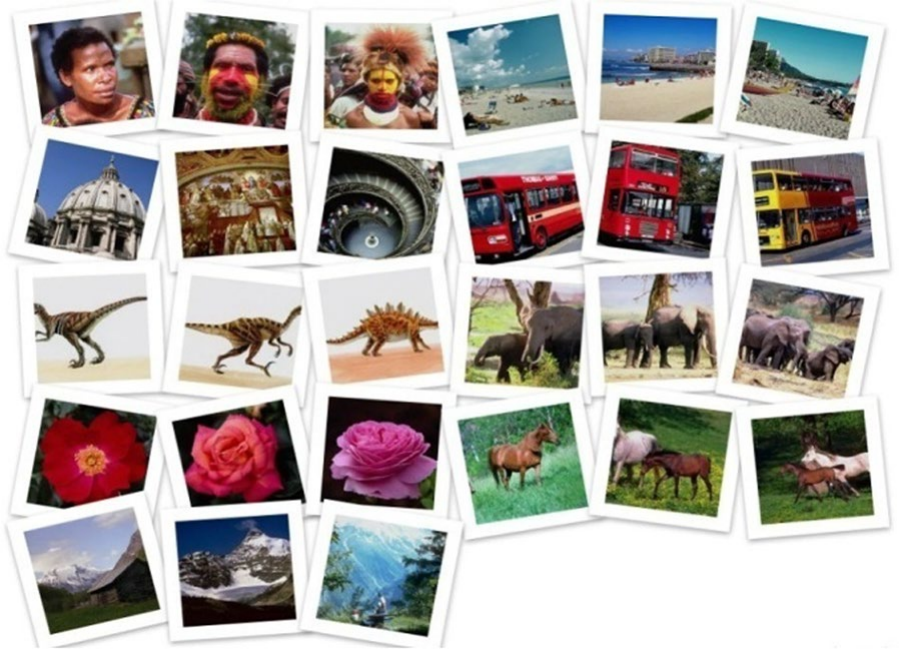
Sample collage for wang dataset

It consists of 10 different categories of 1000 images and was provided by Li and Wang (2003). Each image is of dimension 256 × 384 or 384 × 256 and each category comprises of 100 images. The different classes in this dataset are Tribals, Sea Beaches, Gothic Structures, Buses, Dinosaur, Elephants, Flowers, Horses, Mountains and Food. A sample collage for Wang’s dataset has been given in Fig. 7.

### Oliva and torralba (OT-Scene) dataset

**Fig. 8:**
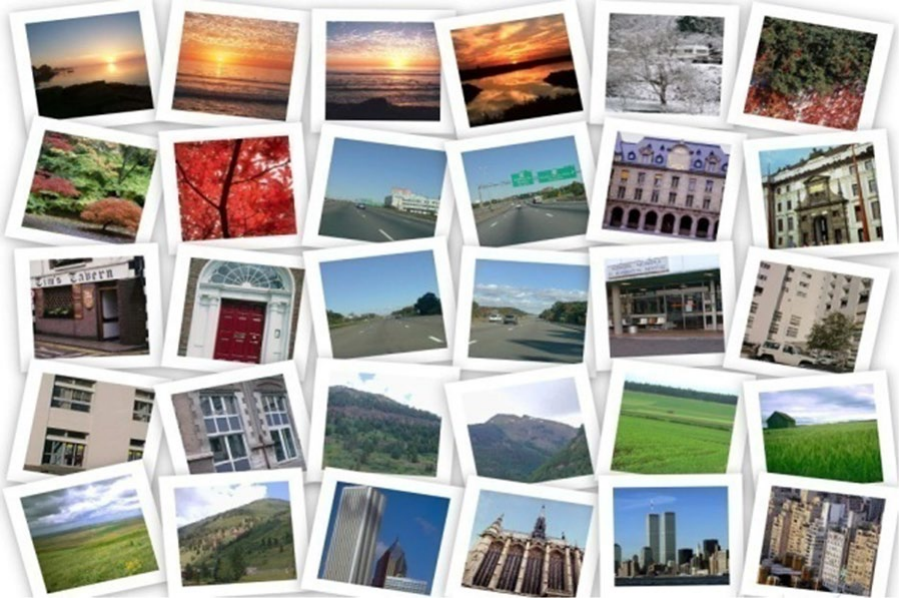
Sample collage for OT-scene dataset

This dataset comprises of 2688 images and is divided into eight different categories. The dataset is provided by MIT (Walia and Pal 2014). The different categories in the dataset are Coast and Beach (with 360 images), Open Country (with 328 images), Forest (with 260 images), Mountain (with 308 images), Highway (with 324 images), Street (with 410 images), City Centre (with 292 images) and Tall Building (with 306 images). A sample collage for OT Scene dataset is given in Fig. 8.

### Corel dataset

**Fig. 9:**
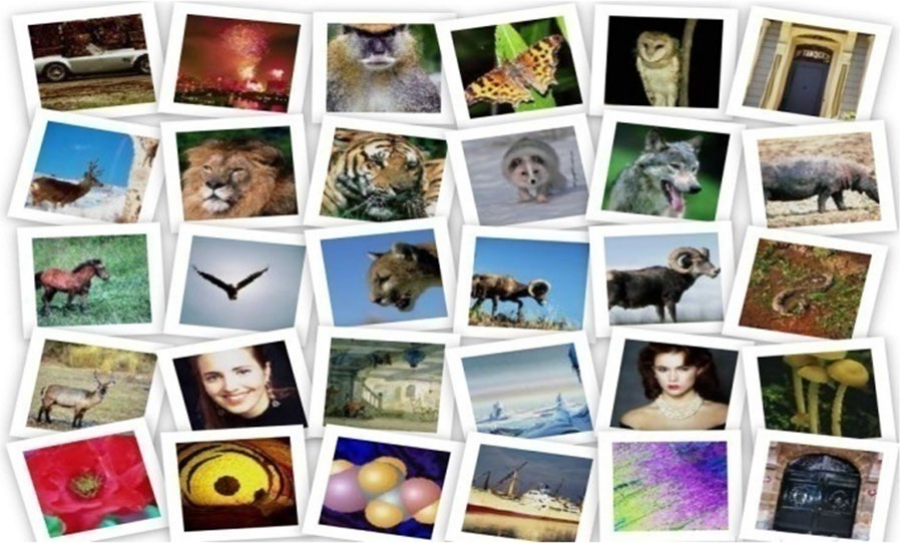
Sample collage for corel dataset

The dataset comprised of 10,800 images (Liu 2013). It has 80 different categories of images of dimension 80 × 120 or 120 × 80. Some of the categories are art, antique, cyber, dinosaur, mural, castle, lights, modern, culture, drinks, feast, fitness, dolls, aviation, balloons, bob, bonsai, bus, car, cards, decoys, dish, door, easter eggs, faces etc. A sample collage of the Corel dataset is given in Fig. 9. The research work has used 2500 images of different categories from this dataset.

### Caltech dataset

**Fig. 10:**
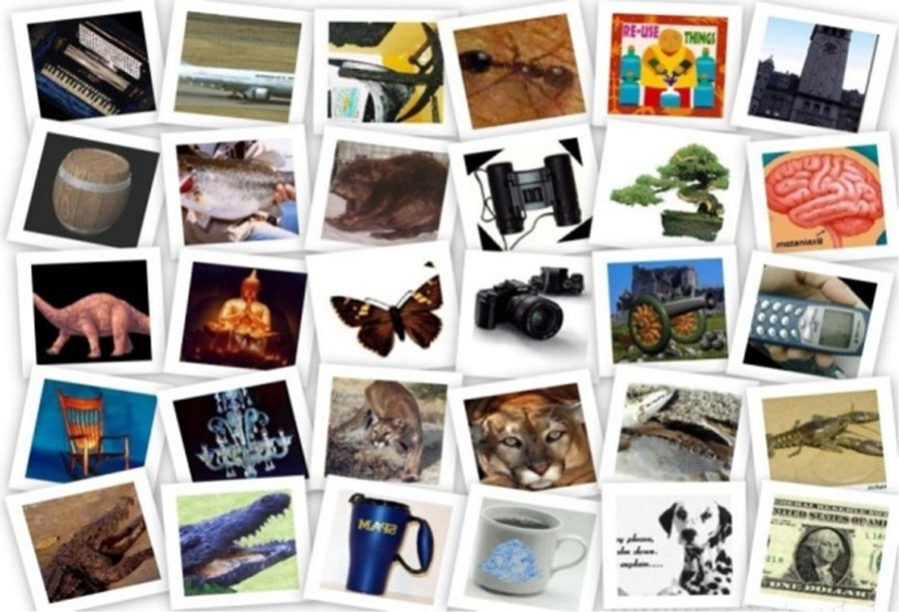
Sample collage for caltech dataset

The dataset includes 8127 images divided into 100 different categories (Walia and Pal 2014). Each of the categories has different number of images with a dimension of 300 x 200. Some of the categories are accordion, airplanes, anchor, ant, Background google, barrel, bass, beaver, binocular, bonsai, brain, brontosaurus, buddha, butterfly, camera, cannon, car side, ceiling fan, cellphone, chair etc. A sample collage for the Caltech dataset has been given in Fig. 10. The research work has used 2533 images of different categories from the dataset.

## Results and discussions

The experiments were executed with Matlab version 7.11.0 (R2010b) on Intel core i5 processor with 4 GB RAM under Microsoft Windows environment. Initially the misclassification rate (MR) and F1 Score for classification with fractional coefficients of slant transform were compared to each other to identify the fractional coefficient with highest classification value and lowest MR. Wang dataset was used for the purpose. Further, the precision and recall values for classification were determined on four different public datasets namely, Wang dataset, OT scene dataset Caltech dataset and Corel dataset. Henceforth, precision and recall values of the fused architecture for classification were compared against state-of-the art techniques. The precision, recall misclassification rate (MR) and F1 Score were represented by Eqs. 16, 17, 18 and 19.

16 $$\Pr ecision = \frac{TP}{TP + FP}$$

17 $$TPRate/\text{Re}\, call = \frac{TP}{TP + FN}$$

18 $$MR = \frac{FP + FN}{TP + TN + FP + FN}$$

19 $$F1score = \frac{{2*\Pr ecision*\text{Re}\, call}}{{\Pr ecision + \text{Re}\, call}}$$

*True Positive (TP)* = *Number of instances classified correctly.True Negative (TN)* = *Number of negative results created for negative instancesFalse Positive (FP)* = *Number of erroneous results as positive results for negative instancesFalse Negative (FN)* = *Number of erroneous results as negative results for positive instances.*

**Fig. 11:**
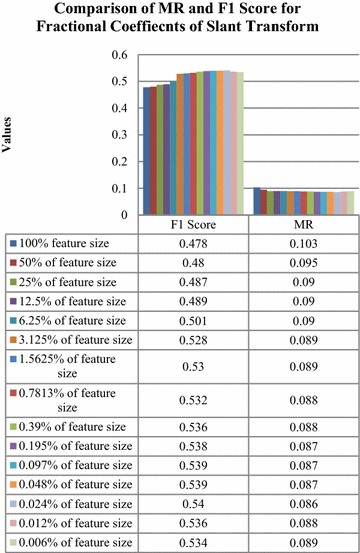
Comparison of MR and F1 score for partial coefficients of slant transform

Comparison of MR and F1 Score for classification with different fractional coefficients of slant transform has been shown in Fig. 11.

It was observed that classification with 0.024 % of the transform coefficient has the highest F1 Score and lowest MR compared to the rest. Hence, it was considered as the feature vector with a dimension of 36.

Further, the precision and recall values of four public datasets have been shown in Table 1.

**Table 1:** Precision and recall values for four public datasets using three feature extraction techniques

	Feature extraction with binarization				Feature extraction with fractional coefficients of slant transform				Feature extraction with morphological operator			
	Wang	OT scene	Caltech	Corel	Wang	OT scene	Caltech	Corel	Wang	OT scene	Caltech	Corel
Precision	0.609	0.41	0.49	0.534	0.555	0.449	0.454	0.527	0.728	0.607	0.523	0.711
Recall	0.604	0.4	0.543	0.519	0.563	0.407	0.523	0.533	0.725	0.597	0.597	0.697

**Fig. 12:**
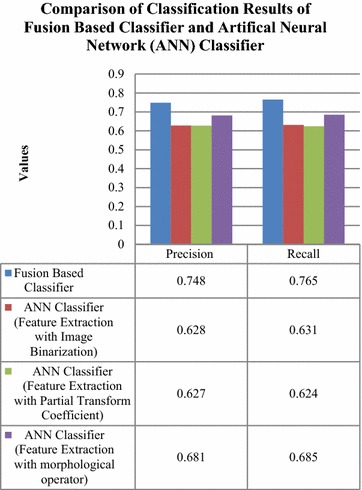
Comparison of classification with fusion based classifier and ANN classifier

Henceforth, Wang dataset was considered in order to carry out classification using fusion technique. The classification decision obtained for Wang dataset using three different feature extraction techniques were fused by means of *Z* score normalization and were compared to classification results obtained by classifying individual techniques by means of artificial neural network (ANN) classifier and support vector machine (SVM) classifier respectively. The comparisons have been shown in Fig. 12.

The comparison in Fig. 12 has clearly revealed that fusion based classification has shown an enhanced precision of 0.12, 0.13 and 0.067 compared to classification with ANN classifier for feature extraction with image binarization, partial transform coefficients and morphological operator respectively. The recall rate for classification with fusion based classification was also higher by 0.134, 0.141 and 0.08 in comparison to classification with ANN classifier for feature extraction with three above mentioned techniques.

**Fig. 13:**
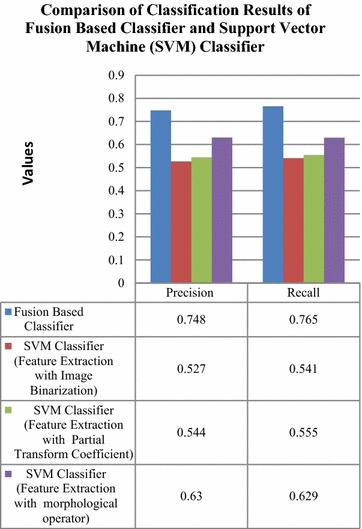
Comparison of classification with fusion based classifier and SVM classifier

The fusion based classifier has revealed an improved precision rate of 0.221, 0.204 and 0.118 in comparison to classification with SVM classifier for feature extraction with image binarization, partial transform coefficient and morphological operator respectively as in Fig. 13. The recall value for classification with fusion based classifier was also higher by 0.224, 0.21 and 0.136 compared to SVM classifier which is seen in Fig. 13.

**Fig. 14:**
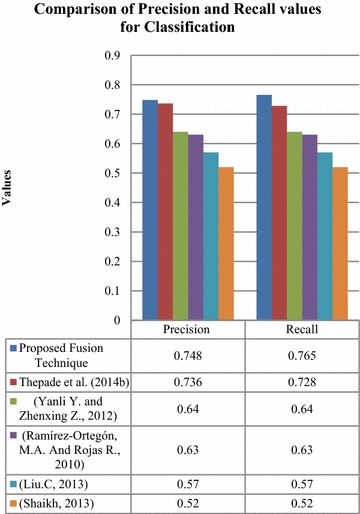
Comparison of classification results of proposed technique with respect to existing techniques

Further, the fusion based classification results were compared to existing techniques in Fig. 14.

It was observed that the proposed method has outclassed the existing techniques. It has an increased precision rate of 0.012, 0.108, 0.109, 0.178 and 0.228 and an enhanced recall rate of 0.037, 0.125, 0.126, 0.195 and 0.245 compared to the existing techniques, namely, (Thepade et al. 2014b; Yanli and Zhenxing 2012; Ramírez-Ortegón and Rojas 2010; Liu 2013; Shaikh et al. 2013) respectively as in Fig. 14. The proposed fusion technique was observed to have the maximum precision and recall values compared to the recent techniques cited in the literature.

**Fig. 15:**
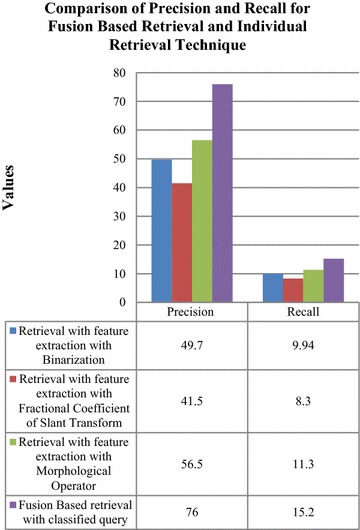
Comparison of precision and recall with fusion based retrieval technique and individual retrieval technique

**Fig. 16:**
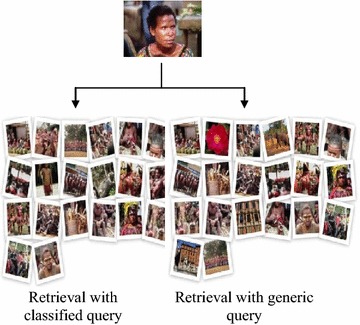
Comparison of fusion based retrieval with classified and generic query

Henceforth, content based image retrieval was carried out with individual techniques of feature extraction and was compared to fusion based technique of retrieval in Fig. 15. The fusion based retrieval technique comprised of classification as a precursor of retrieval. Comparison of fusion techniques with classified query and without classified query has been shown in Fig. 16 by using a sample query.

The figure has clearly divulged that fusion technique of retrieval with classified query has fetched all the images of the same category to that of the query image, whereas, retrieval with generic or unclassified query has three images from classes other than the class of query in position 2, 15 and 19 respectively.

A comparison of retrieval with individual techniques of feature extraction and fusion based retrieval with classified query has been given in Fig. 15.

Results in Fig. 15 have shown an increase of 26.3, 34.5 and 19.5 % in precision values and enhancement of 5.26, 6.9 and 3.9 % in recall values for the fusion based retrieval technique with classified query in comparison to retrieval with individual feature extraction techniques. It was clearly established that the fusion based technique has outperformed the individual techniques.

Further, a paired *t* test was conducted to validate the statistical findings and a null hypothesis was formulated in Hypothesis 1 (Yıldız et al. 2011).

*Hypothesis 1*: *There is no significant difference among the Precision values of fusion based retrieval with classified query with respect to individual retrieval techniques*

The *p* values for the paired *t* test have been enlisted in Table 2. The precision value of fusion based retrieval with classified query was compared to that of the individual retrieval techniques to obtain the computed values in Table 2.

**Table 2:** Statistical validation with paired *t* test

	p value	Significance
Retrieval by feature extraction with image transform	0.0013	Significant
Retrieval by feature extraction with image binarization	0.0076	Significant
Retrieval by feature extraction with morphological operator	0.0452	Significant

The *p* values have clearly indicated significant difference in precision values of the fusion based retrieval technique with classified query compared to the existing techniques of retrieval. Hence, the null hypothesis was rejected and the proposed fusion technique with classified query has been found to boost the precision values with statistical significance.

**Fig. 17:**
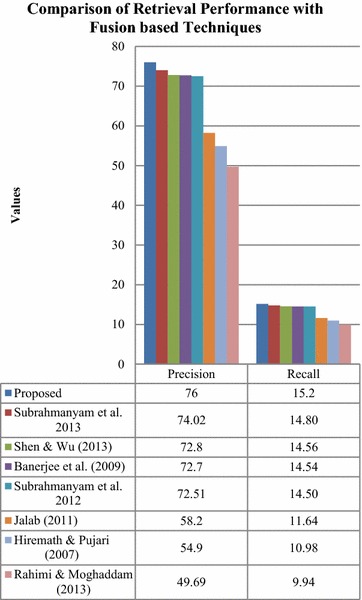
Comparison of retrieval with the proposed technique compared to state-of-the art fusion techniques

Finally, the precision and recall values of the proposed fusion technique were compared to existing fusion based retrieval techniques. The results have been displayed in Fig. 17.

The comparison in Fig. 17 has clearly established the superiority of the proposed fusion based retrieval technique with respect to existing fusion based technique of retrieval. The proposed retrieval technique has improved precision of 1.98, 3.2, 3.3, 3.49, 17.8, 21.1 and 26.31 % and superior recall of 0.4, 0.64, 0.66, 0.7, 3.56, 4.22 and 5.26 % compared to the existing fusion based techniques mentioned in Fig. 13.

**Fig. 18:**
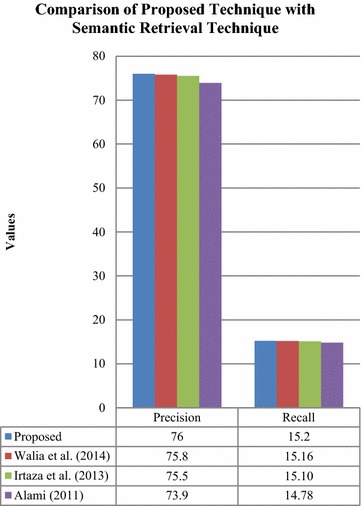
Comparison of retrieval with the proposed technique to semantic retrieval techniques

Henceforth, the proposed method was compared to the semantic retrieval techniques in Fig. 18.

The comparison shown in Fig. 18 has revealed an enhanced precision rate of 0.2, 0.5 and 2.1 % and increased recall rate of 0.04, 0.1 and 0.6 % respectively for the proposed method with respect to the existing semantic retrieval techniques.

Therefore, the research work has fulfilled the following objectives:

- It has reduced the dimension of feature vectors.
- It has successfully implemented fusion based method of content based image identification.
- The research results have shown statistical significance.
- The research results have outperformed the results of state-of-the art techniques.

## Conclusions

In depth analysis of feature extraction techniques have been exercised in this research work. Three different techniques of feature extraction comprising of image binarization, fractional coefficients of image transforms and morphological operations has been implemented to extract features from the images. The extracted features with multiple techniques were used for fusion based identification process. The proposed method of fusion has divulged statistical significance with respect to the individual techniques. The retrieval technique was implemented with classification as a precursor. The classification technique was used to classify the query image for retrieval. The method has shown better performance compared to generic query based method of retrieval. Thus, the importance of classification was established in limiting the computational overhead for content based image identification. Finally, image identification with the proposed technique has surpassed the state-of-the art methods for content based image recognition. The work may be extended towards content based image recognition in the field of military, media, medical science, journalism, e commerce and many more.

## References

[CR1] Alsmadi MK, Omar KB, Noah SA, Almarashdah I (2009) Performance comparison of multi-layer perceptron (Back Propagation, Delta Rule and Perceptron) algorithms in neural networks. 2009 IEEE International Advance Computing Conference, IACC 2009, 7: pp 296–299

[CR2] Annadurai S, Shanmugalakshmi R (2011) Image transforms, fundamentals of digital image processing. Dorling Kindersley (India) Pvt. Ltd., pp 31–66

[CR3] Banerjee M, Kundu MK, Maji P (2009). Content- based image retrieval using visually significant point features. Fuzzy Sets Syst.

[CR4] Douze M, Jégou H, Singh H, Amsaleg L, Schmid C (2009) Evaluation of GIST descriptors for web-scale image search. In ACM International Conference on Image and Video Retrieval, pp 0–7

[CR5] Dubois SR, Glanz FH (1986) An autoregressive model approach to two-dimensional shape classification. IEEE Trans Pattern Anal Mach Intell 8(1):55–66 10.1109/tpami.1986.4767752 21869323

[CR6] Dunham MH (2009) Data Mining Introductory and Advanced Topics: Pearson Education, p 127

[CR7] ElAlami ME (2011). A novel image retrieval model based on the most relevant features. Knowl-Based Syst.

[CR8] ElAlami ME (2014). A new matching strategy for content based image retrieval system. Appl Soft Comput J.

[CR9] Flickner M, Sawhney H, Niblack W, Ashley J, Huang Q, Dom B, Gorkani M (1995). Query by image and video content: the QBIC system. Computer.

[CR10] Gevers T, Smeulders AW (2000). PicToSeek: combining color and shape invariant features for image retrieval. IEEE Trans Image Proc Publ IEEE Signal Proc Soc.

[CR11] Hiremath PS, Pujari J (2007). Content based image retrieval based on color, texture and shape features using image and its complement. Int J Computer Sci Secur.

[CR12] Irtaza A, Jaffar MA, Aleisa E, Choi TS (2013) Embedding neural networks for semantic association in content based image retrieval. Multimed Tool Appl 72(2):1911–1931

[CR13] Jalab HA (2011) Image retrieval system based on color layout descriptor and Gabor filters. 2011 IEEE Conference on Open Systems. pp 32–36

[CR14] Keerthi SS, Shevade SK, Bhattacharyya C, Murthy KRK (2001). Improvements to Plattʼs SMO Algorithm for SVM classifier design. Neural Comput.

[CR15] Kekre HB, Thepade S (2009). Improving the performance of image retrieval using partial coefficients of transformed image. Int J Inf Retr Ser Publ.

[CR16] Kekre HB, Thepade S, Maloo A (2010). Image Retrieval using Fractional Coefficients of Transformed Image using DCT and Walsh Transform‖. Int J Eng Sci Technol (IJEST).

[CR17] Kekre HB, Thepade S, Das R, Ghosh S (2013) Multilevel block truncation coding with diverse colour spaces for image classification. In: IEEE-International conference on Advances in Technology and Engineering (ICATE), pp 1–7

[CR18] Kim WY, Kim YS (2000). Region-based shape descriptor using Zernike moments. Sig Process Image Commun.

[CR19] Li J, Wang JZ (2003). Automatic linguistic indexing of pictures by a statistical modeling approach. IEEE Trans Pattern Anal Mach Intell.

[CR20] Liu C (2013) A new finger vein feature extraction algorithm, In: IEEE 6th. International Congress on Image and Signal Processing (CISP), pp 395–399

[CR21] Luo H, Lina Y, Haoliang Y, Yuan YT (2013) Dimension reduction with randomized anisotropic transform for hyperspectral image classification. In: 2013 IEEE International Conference on Cybernetics, CYBCONF 2013, pp 156–161

[CR22] Madireddy RM, Gottumukkala PSV, Murthy PD, Chittipothula S (2014). A modified shape context method for shape based object retrieval. SpringerPlus.

[CR23] Mehtre BM, Kankanhalli MS, Lee Wing Foon (1997). Shape measures for content based image retrieval: a comparison. Inf Process Manage.

[CR24] Mokhtarian F, Mackworth AK (1992). A theory of multiscale, curvature-based shape representation for planar curves. IEEE Trans Pattern Anal Mach Intell.

[CR25] Otsu N (1979). A threshold selection method from gray- level histogram IEEE transactions on systems. Man Cybern.

[CR26] Prakash O, Khare M, Srivastava RK, Khare A (2013) Multiclass image classification using multiscale biorthogonal wavelet transform, In: IEEE Second International Conference on Information Processing (ICIIP), pp 131–135

[CR27] Pratt W, Chen WH, Welch L (1974) Slant transform image coding. IEEE Transactions on Communications 22

[CR28] Rahimi M and Moghaddam ME (2013) A content based image retrieval system based on color ton distributed descriptors. Signal Image Video Process 9(3):691–704. http://dx.doi.org/10.1007/s11760-013-0506-6

[CR29] Ramírez-Ortegón MA and Rojas R (2010) Unsupervised evaluation methods based on local gray-intensity variances for binarization of historical documents. Proceedings—International Conference on Pattern Recognition, pp 2029–2032

[CR30] Raventós A, Quijada R, Torres L, Tarrés F (2015). Automatic summarization of soccer highlights using audio- visual descriptors. SpringerPlus.

[CR31] Shaikh SH, Maiti AK, Chaki N (2013). A new image binarization method using iterative partitioning. Mach Vis Appl.

[CR32] Shen GL and Wu XJ (2013) Content based image retrieval by combining color texture and CENTRIST, In: IEEE international workshop on signal processing, vol 1, pp 1–4

[CR33] Sridhar S (2011). Image features representation and description digital image processing.

[CR34] Subrahmanyam M, Maheshwari RP, Balasubramanian R (2012). Expert system design using wavelet and color vocabulary trees for image retrieval. Expert Syst Appl.

[CR35] Subrahmanyam M, Wu QMJ, Maheshwari RP, Balasubramanian R (2013). Modified color motif co- occurrence matrix for image indexing and retrieval. Comput Electr Eng.

[CR36] Thepade S, Das R, Ghosh S (2013). Advances in computing, communication and control. Image classification using advanced block truncation coding with ternary image maps.

[CR37] Thepade S, Das R, Ghosh S (2013b) Performance comparison of feature vector extraction techniques in RGB color space using block truncation coding or content based image classification with discrete classifiers. In: India Conference (INDICON), IEEE, pp 1–6. doi: 10.1109/INDCON.2013.6726053

[CR38] Thepade S, Das R, Ghosh S (2014a) A novel feature extraction technique using binarization of bit planes for content based image classification. J Eng. doi:10.1155/2014/439218

[CR01] Thepade S, Das R, Ghosh S (2014b) Feature extraction with ordered mean values for content based image classification. Adv Comput Eng 2014. doi:10.1155/2014/454876

[CR39] Valizadeh M, Armanfard N, Komeili M, Kabir E (2009) A novel hybrid algorithm for binarization of badly illuminated document images. 2009 14th International CSI Computer Conference, CSICC 2009, pp 121–126

[CR40] Walia E, Pal A (2014) Fusion framework for effective color image retrieval. J Vis Commun Image Represent 25(6):1335–1348

[CR41] Walia E, Vesal S, Pal A (2014) An Effective and Fast Hybrid Framework for Color Image Retrieval. Sens Imaging 15:93. doi: 10.1007/s11220-014-0093-9

[CR42] Wang X, Bian W, Tao D (2013). Grassmannian regularized structured multi-view embedding for image classification. IEEE Trans Image Process.

[CR43] Yanli Y and Zhenxing Z (2012) A novel local threshold binarization method for QR image, In: IET International Conference on Automatic Control and Artificial Intelligence (ACAI), pp 224–227

[CR44] Yıldız OT, Aslan O, Alpaydın E (2011) Multivariate statistical tests for comparing classi-fication algorithms. Lect Notes Comp Sci, vol 6683, Springer, Berlin, pp 1–15

[CR45] Yue J, Li Z, Liu L, Fu Z (2011). Content-based image retrieval using color and texture fused features. Math Comput Model.

[CR46] Zhang D, Lu G (2003). A comparative study of curvature scale space and Fourier descriptors for shape- based image retrieval. J Vis Commun Image Represent.

[CR47] Zhang D, Lu G (2004). Review of shape representation and description techniques. Pattern Recogn.

[CR48] Zhao C, Li X, Cang Y (2015) Bisecting k-means clustering based face recognition using block-based bag of words model. Optik Int J Light Electron Optics 126(19):1761–1766

[CR49] Zhu Q, Shyu M-L (2015). sparse linear integration of content and context modalities for semantic concept retrieval. IEEE Trans Emerg Top Comput.

